# Toll receptor ligand Spätzle 4 responses to the highly pathogenic *Enterococcus faecalis* from *Varroa* mites in honeybees

**DOI:** 10.1371/journal.ppat.1011897

**Published:** 2023-12-27

**Authors:** Wenhao Zhang, Cheng Sun, Haoyu Lang, Jieni Wang, Xinyu Li, Jun Guo, Zijing Zhang, Hao Zheng

**Affiliations:** 1 Faculty of Food Science and Engineering, Kunming University of Science and Technology, Kunming, China; 2 College of Life Sciences, Capital Normal University, Beijing, China; 3 College of Food Science and Nutritional Engineering, China Agricultural University, Beijing, China; 4 Faculty of Life Science and Technology, Kunming University of Science and Technology, Kunming, China; 5 Hebei Key Laboratory of Animal Physiology, Biochemistry and Molecular Biology, Hebei Collaborative Innovation Center for Eco-Environment, College of Life Sciences, Hebei Normal University, Shijiazhuang, China; Pennsylvania State University - Main Campus: The Pennsylvania State University - University Park Campus, UNITED STATES

## Abstract

Honeybees play a major role in crop pollination, which supports the agricultural economy and international food supply. The colony health of honeybees is threatened by the parasitic mite *Varroa destructor*, which inflicts physical injury on the hosts and serves as the vector for variable viruses. Recently, it shows that *V*. *destructor* may also transmit bacteria through the feeding wound, yet it remains unclear whether the invading bacteria can exhibit pathogenicity to the honeybees. Here, we incidentally isolate *Enterococcus faecalis*, one of the most abundant bacteria in *Varroa* mites, from dead bees during our routine generation of microbiota-free bees in the lab. *In vivo* tests show that *E*. *faecalis* is only pathogenic in *Apis mellifera* but not in *Apis cerana*. The expression of antimicrobial peptide genes is elevated following infection in *A*. *cerana*. The gene-based molecular evolution analysis identifies positive selection of genes encoding Späetzle 4 (Spz4) in *A*. *cerana*, a signaling protein in the Toll pathway. The amino acid sites under positive selection are related to structural changes in Spz4 protein, suggesting improvement of immunity in *A*. *cerana*. The knock-down of *Spz4* in *A*. *cerana* significantly reduces the survival rates under *E*. *faecalis* challenge and the expression of antimicrobial peptide genes. Our results indicate that bacteria associated with *Varroa* mites are pathogenic to adult bees, and the positively selected gene *Spz4* in *A*. *cerana* is crucial in response to this mite-related pathogen.

## Author summary

Multiple factors contribute to the decline of honeybee colonies, including climate change, the spread of pests and pathogens, and pesticide use. *Varroa* mites and the viruses they carry pose a severe threat to honeybee health. However, it is still unknown whether the bacteria from *Varroa* mites are also responsible for the death of worker bees. Indeed, these bacterial pathogens were underestimated since infected adult workers tend to abandon their hives. Here, we characterized the virulence of *E*. *faecalis* strains isolated from the gut of *A*. *mellifera* originating from mite-infected hives. Our results indicate that *E*. *faecalis*, an opportunistic pathogen of many plants and animals, poses a significant threat to *A*. *mellifera* but not *A*. *cerana*. Infection of *E*. *faecalis* stimulates the Toll signaling pathway and the downstream expression of AMPs in *A*. *cerana*. Specifically, Spz4 protein has been selected in a relatively longer evolutionary process in *A*. *cerana*, and is the key mechanism of *A*. *cerana* to defend against *E*. *faecalis*. Our results suggest that eastern and western honeybees may possess different innate immune responses against the *V*. *destructor*-associated bacterial pathogens.

## Background

Honeybees are important crop pollinators worldwide supporting the global food supply and the agricultural economy, but their population has declined dramatically in recent years. Multiple factors threaten honeybee health, such as pesticide application, habitat loss, parasites, and pathogens [1]. The ectoparasitic mite *Varroa destructor* is one of the major threats to Western honeybees (*Apis mellifera*) [2–4]. Importantly, *Varroa* mites vector many honeybee viruses, causing honeybee health to decline and eventually leading to colony death [5–7]. *V*. *destructor* mites were initially found restricted to the Eastern honeybees (*Apis cerana*) and spread to *A*. *mellifera* in the first half of the 20th century [8]. Compared to the Western honeybee, *A*. *cerana* exhibits natural resistance to *Varroa* mites [9]. Several strategies applied by *A*. *cerana* make it less vulnerable to *V*. *destructor* infection, such as preventing reproduction in worker hives [10], burying immature drones [11], and efficient grooming and hygienic behavior in adult worker bees [12]. Furthermore, *A*. *cerana* tends to produce a higher diversity of antimicrobial peptides (AMPs) than *A*. *mellifera*, which aids in its defense against pathogens [13,14].

In addition to viruses, a high level of *Varroa* infestation is often accompanied by increased incidences of bacteria within a domesticated honeybee colony [15]. *V*. *destructor* carries a wide variety of bacteria, including *Enterococcus faecalis*, *Bacillus subtilis*, *Pseudomonas syringae*, *Terribacillus goriensis*, and *Morganella* spp. [15–18]. *E*. *faecalis* is a well-known opportunistic pathogen that causes more than half of the mortality rate after infection in many animals, especially for insects, such as honeycomb moth (*Galleria mellonella*) [19] and *Drosophila* [20]. *E*. *faecalis* from the mites has been detected in bee brood and mite-infected adult bees in Kenya [15]. This bacterium has also been identified as highly abundant in worker bees from colonies with European foulbrood, and confirmed as a secondary invader in septicemic infections in *A*. *mellifera* [21]. A recent study demonstrated that *Varroa* mites can transmit certain bacteria into worker bees when consuming their fat bodies [22], and the invading bacteria can exhibit high virulence once enter the hemolymph of bees [23,24].

In this study, during our routine generation of microbiota-free (MF) honeybees in the lab, we ran into an unexpectedly high mortality rate of the bees. We found the brood frames we used were from *Varroa* mite-infested colonies. The dead bees harbored abundant non-core bacteria, specifically *E*. *faecalis*, in their guts. We isolated *E*. *faecalis* zzj01 from the gut of dead MF bees, and this strain exhibits high virulence to *A*. *mellifera* but has little effect on the lethality of *A*. *cerana*. In addition, the gene expression of AMPs was induced by *E*. *faecalis* zzj01 only in *A*. *cerana*. The evolutionary analysis revealed that *Spz4* is the positive selection gene in *A*. *cerana*. RNA interference experiments confirmed that *Spz4* plays a key role in AMP production in *A*. *cerana* and is crucial for the resistance to *E*. *faecalis*.

## Methods

### Isolation and genome sequencing of the pathogenic *E*. *faecalis* from dead bees

To understand the reason for the abnormally elevated mortality rate in MF bees, *E*. *faecalis* strains were isolated from the guts of dead MF bees generated from a *V*. *destructor*-infested *A*. *mellifera* colony in Yunnan, China, in July 2020. The dissected guts were directly crushed in PBS after sampling. The PBS stocks were plated on brain heart infusion agar (BHI) supplemented with 5% (vol/vol) defibrinated sheep’s blood (Solarbio, Beijing, China) and incubated at 35°C under a CO_2_-enriched atmosphere (5%). After 24-hour incubation, bacterial colonies from different plates were selected and restreaked consecutively three times to ensure purity.

The isolated bacteria were identified by PCR with universal bacterial primers 27F (5’-AGAGTTTGATCCTGGCTCAG-3’) and 1492R (5’-TACGACTTAACCCCAATCGC-3’). Sanger sequencing was performed at Sangon Biotech Co. Ltd., Shanghai, China. Bacterial isolates were identified using a 16S sequence blast against the NCBI database, showing>97% similarity with *E*. *faecalis* strain HBUR51110. The genomic DNA of *E*. *faecalis* strain zzj01 was extracted using a Bacterial Genomic DNA Extraction kit (Tiangen, Beijing, China). Whole genomic DNA was sequenced on the Illumina HiSeq platform with paired-end libraries and then assembled with the SPAdes genome assembler (version 3.0) [25]. The completeness of the draft genomes was assessed by CheckM (version 1.0.12) [26]. The whole-genome average nucleotide identity between *E*. *faecalis* GCF 005484525.1 and *E*. *faecalis* GCF 014489455.1 was calculated using FastANI (version 2.0) [27].

### Phylogenomic analysis of the isolated *E*. *faecalis* strains

The whole genome sequences of all available *E*. *faecalis* strains were collected from the NCBI Refseq database (June 2021), as well as the genome of *Melissococcus plutonius* strain DAT561 (GCF_003966875.1), which was used as an outgroup. All genomes were annotated with the Prokka software (version 1.14.0) [28]. Usearch (version9.0) [29] was used to identify orthologs across *E*. *faecalis* strains (based on best reciprocal hits) with > 70% sequence identity and > 80% sequence length conservation. Single-copy gene families were detected in all examined genomes, then aligned and concatenated using MAFFT (version 7) [30]. The concatenated alignment was used to build a maximum likelihood tree using PhyML (version 3.0) [31] with the following parameters: GTR, Gamma4, and 100 bootstrap replicates.

### Survival assay of honeybees after *E*. *faecalis* treatment

To verify the virulence of the isolated *E*.*faecalis* strain to honeybees, we conducted a feeding exposure experiment with *E*.*faecalis* on *A*. *mellifera* and *A*. *cerana*. All bees were obtained from mite-free colonies maintained in the experimental apiary of the Kunming University of Science and Technology. Microbiota-free (MF) bees were generated in the lab, as described by Zheng et al. [32]. In brief, late-stage pupae were removed from brood frames using sterilized tweezers and placed in sterile plastic boxes. The pupae emerged in an incubator at 35 °C, with a humidity of 50%. Newly emerged MF bees (Day 0) were kept in axenic cup cages for 24 hours and supplied with sterilized sucrose syrup (50%, wt/vol). MF bees (Day 1) were then divided into two groups: 1) MF and 2) gnotobiotic bees with a conventional (CV) gut microbiota. Approximately 20–25 MF bees (Day 1) were placed in one cup cage for each group and fed the respective liquids or suspension for 24 hours. For the MF group, 1 ml of 1×PBS was mixed with 1 ml of sterilized sucrose solution (50%, wt/vol) and 0.3 g sterilized pollen. For the CV group, 5 μl homogenates of freshly dissected hindguts of healthy worker bees from the hives were mixed with 1 ml 1×PBS, 1 ml sterilized sucrose solution (50%, wt/vol), and 0.3 g sterilized pollen.

To demonstrate the effect of *E*. *faecalis* on the host, MF bees (Day 0) were divided into two groups: 1) MF (control) and 2) MF+*E*. *faecalis*. Each group was divided into three cup cages containing approximately 20–25 bees, feeding on the corresponding solutions or suspensions for 24 hours. Bees in the control (MF) group were generated as MF bees described above. For the MF+*E*. *faecalis* group, stock of *E*. *faecalis* in 25% glycerol stock at -80°C was resuspended in 1 ml 1×PBS at a final OD_600nm_ of 1.0 and then mixed with 1  ml sterilized sucrose solution (0.5 M) and 0.3 g sterilized pollen.

To test the protective effect of gut microbiota against *E*. *faecalis* infection, MF and CV bees (Day 5) were divided into three groups: 1) CV (control), 2) CV+*E*. *faecalis*, and 3) MF+*E*. *faecalis*. Each group contains 20–25 bees with three replicate cups. Bees in the control (CV) group were generated as CV bees described above. For the CV+*E*. *faecalis* and MF+*E*. *faecalis* groups, 5-day-old CV bees, and 5-day-old MF bees were fed with *E*. *faecalis* suspension (OD_600nm_ of 1.0) for 24 hours, then replaced with sterilized sucrose (0.5 M) and pollen.

### Hemolymph injections of *E*. *faecalis*

Cell suspension of *E*. *faecalis* (OD_600nm_ = 1) in PBS was 10x serial diluted to 10^−4^, and 1 μl of each diluted solution was injected into the abdomen of MF bees using a microliter syringe (Shanghai Bolige Industry & Trade, ⌀ = 0.26 mm). Bees in the control group were injected with 1  μl of sterile PBS. Each group was divided into four cup cages with about 12 bees in each cup. The survival of the bees was then monitored for 24 hours, and the number of dead bees for each treatment group was counted every 2 hours. Bees were placed in an incubator at 35°C and 95% relative humidity to simulate hive conditions during the experiments.

### Quantitative PCR determining immune response to *E*. *faecalis* infection

One-day-old MF bees were mono-infected with *E*. *faecalis*. After 24-hour colonization, whole guts were dissected and transferred to an RNase-free 1.5-ml tube. Total RNA was extracted using the FassPure Cell/Tissue Total RNA Isolate Kit V2 (Vazyme, Nanjing, China). cDNA was then synthesized using the HiScript III RT SuperMix for qPCR (Vazyme) according to the manufacturer’s protocols. cDNA samples were diluted (1:10) in Rnase-free water for quantitative PCR. The primers of the immune genes are listed in S1 Table. All qPCRs were performed using the Taq pro Universal SYBR qPCR Master Mix (Vazyme) and CFX96 Touch Real-Time PCR Detection System (Bio-Rad, Hercules, CA, USA) in a standard 96-well block. Melting curves were generated after each run (95°C for 15 s, 60°C for 20 s, and increments of 0.3°C until reaching 95°C for 15 s). Expression levels were measured in triplicate for each biological replicate and normalized against the housekeeping *actin* or *RPS18* gene. The relative expression level of genes was calculated using the 2^-ΔΔCT^ method [33].

### Detection of positive selection on immune genes in honeybees

To investigate the evolution of genes in *A*. *cerana*, the genome of five *Apis* species (*Apis dorsata*, *Apis florea*, *Apis laboriosa*, *A*. *mellifera*, *A*. *cerana*) and *B*. *terrestris* were selected for evolutionary analysis. Nonsynonymous (*dN*) and synonymous (*dS*) are used to estimate the evolutionary rate and positively selected genes (PSGs) in each species. To eliminate biases caused by lineage duplication and out-paralog genes, only universal single-copy orthologous groups (scOGs) were utilized to calculate the *dN/dS* ratios of *Apis* species and *B*. *terrestris*. Protein sequences of scOGs were aligned using MAFFT (version 7) [30]. The resulting CDS alignments were converted into DNA codon alignments by the codon-aware PAL2NAL program (version 13) [34]. The aligned CDSs were then trimmed by Gblocks (version 0.91) [35]. Next, RaxML-NG [36] was used to build Maximum Likelihood trees for each orthologous group (*Apis* species and *B*. *terrestris*) based on the trimmed alignments. Finally, the phylogenetic tree was used to calculate the *dN/dS* ratio for each orthologous group (codeml model = 1, Nssites = 0) by *PAML* [37].

To identify immune-related genes involved in adaptation to pathogens, searches were conducted for genes undergoing positive selection in *A*. *cerana* and *A*. *mellifera*. Universal single-copy orthologous groups and their respective multiple sequence alignments and Maximum Likelihood trees were obtained. Then, genes showing signatures of positive selection were identified by the improved branch-site model in the Codeml program of the *PAML* package [38]. *A*. *cerana* was assigned as the foreground branches, whereas the other *Apis* species (*A*.*dorsata*, *A*. *florea*, *A*. *laboriosa*, *A*. *mellifera*) and *B*. *terrestris* were the background branches. A positive selection model that allowed a class of codons on the foreground branches to have *dN/dS* > 1 (model = 2, Nssites = 2, omega = 0.5|1.5, fix_omega = 0) was compared with a null model that constrained this class of sites to have *dN/dS* = 1 (model = 2, Nssites = 2, omega = 1, fix_omega = 1) using a likelihood ratio test and calculated a p-value for each comparison. Multiple comparisons were corrected using the Benjamini and Hochberg method and selected genes with an adjusted p-value < 0.05 as candidate positively selected genes (PSGs). The Bayes Empirical Bayes (BEB) method [39] was utilized to calculate posterior probabilities for site classes, identifying codon positions that underwent positive selection. Finally, Codeml estimated the dN, dS, and *dN/dS* of these PSGs with the free ratio model (model = 1, Nssites = 0). PSGs with dS >1 indicating considerable saturation at synonymous sites were removed from downstream analysis to avoid false positives.

To validate the positively selected sites identified by PAML, we implemented an alternative algorithm designed to cross-verify the selection signatures. The aligned codons sequences of the *Spz4* gene in *Apis* species and *B*. *terrestris* were analyzed using the Datamonkey web server (https://www.datamonkey.org), with the Fixed Effects Likelihood (FEL) to detect positive selection (*A*. *cerana* and *A*. *mellifera* as the test branches and others as background). Orthologous groups containing focal genes and their *dN/dS* values were extracted from the Molecular evolution analysis in the gene functional categories section. Only universal single-copy orthologous groups were kept for downstream analysis. The multiple alignments and Maximum Likelihood tree of each ortholog were obtained. Then, the aligned codon sequences of the *Spz4* gene in various *Apis* species and *B*. *terrestris* were uploaded to the Datamonkey server. The FEL method is run on the server to estimate the *dN/dS* ratio at each site in the gene sequence. Sites with a high *dN/dS* ratio and a significant p-value (< 0.05) were considered under positive selection.

### RNA interference of *Spz4* and survival assay

We carried out RNA interference (RNAi) experiments to examine the role of the *Spz4* gene in the innate immunity of *A*. *cerana*. The total RNA of each dissected gut was extracted, and cDNA was transferred as described above. To produce the double-stranded RNA (dsRNA) of the *Spz4* and *Spz5* (control) genes, the coding regions of genes were amplified from *A*. *cerana* cDNA using specific primers with T7 promoter sequence at their 5’ ends. The primers were designed based on the nucleotide sequences available in GenBank: spaetzle 4 (*Spz4*) (XM_028668966.1): forward 5′-CAACGAATTCAGGGACGAGG-3′, reverse 5′-AGTAGTGCCGGGGAAATTCA-3′; spaetzle 5 (*Spz5*) (XM_028665600.1): forward 5′-CAACCTTTGAACGCCTTTGG-3′, reverse 5′-ATTCAAAGCTGCTCTCGGTG-3′. Then, the partially amplified segments of *Spz4* and *Spz5* were cloned into the pCE2 TA/Blunt-Zero vector (Vazyme) and verified by Sanger sequencing. The FastPure Plasmid Mini Kit (Vazyme) was used to isolate *E*. *coli* plasmid DNA. During cloning procedures, DNA fragments were purified by FastPure Gel DNA Extraction Mini Kit (Vazyme). Finally, the fragment was amplified from the plasmid using specific primers with a T7 promoter and then used for dsRNA synthesis by the T7 RNAi Transcription Kit (Vazyme). The star polycation was used as a gene nanocarrier to protect dsRNA molecules from degradation and promote translocation [40]. Nanocarriers were mixed gently with dsRNA at a recommended mass ratio of 1:1 to improve the efficiency of RNAi in feeding and then diluted with nuclease-free water to a final concentration of 2 μg/μl.

To determine whether the *Spz4* gene enhances the immune response of *A*. *cerana*, bees were randomly divided into three groups (*E*. *faecalis*, *E*. *faecalis*+ds*Spz4*, *E*. *faecalis*+ds*Spz5*) for RNAi experiments and survival assay. Newly emerged MF bees in the *E*. *faecalis*+ds*Spz4* and *E*. *faecalis*+ds*Spz5* groups were fed with sucrose solution (0.5 M) containing dsSpz4 or dsSpz5 and ingested about 20 μg of dsRNA per day. The *E*. *faecalis* group was only supplied with sucrose water solution (0.5 M) as the untreated control. After two days, the gene expression levels of *Spz4* and *Spz5* were quantified with qPCR. Compared to untreated controls, a 70% reduction in the relative expression was considered an effective silencing of target genes. Then, bees in all groups were inoculated with *E*. *faecalis*, as described above. After 24-hour exposure, the relative expression of *abaecin*, *apidaecin*, *defensin-1*, *defensin-2*, *hymenoptaecin*, and *lysozyme* was analyzed by qPCR. Finally, the mortality of bees in each group was recorded every day for seven days.

## Results

### *E*. *faecalis* is pathogenic in *A*. *mellifera* but not in *A*. *cerana*

During the generation of MF bees in the laboratory in July 2020, we encountered an abnormally high mortality in the newly emerged MF bees. We observed an unexpected infestation of *V*. *destructor* in some of the honeycombs when collecting samples from domesticated *A*. *mellifera* hives from our apiary in Kunming. To determine the sterility of the bees, we spread the diluted homogenate of the MF bee gut onto BHI agar plates. We observed white and somewhat translucent bacterial colonies on the BHI plates, indicating that these MF bees were not axenic (bacterial load > 10^4^/gut). We randomly picked four single colonies, and they were identified as *E*. *faecalis* with identical 16S rRNA sequences. We chose one strain, *E*. *faecalis* zzj01, for whole genome sequencing. Phylogenetic and average nucleotide identity (ANI) analysis confirmed that the strain belongs to the species *E*. *faecalis* (S1 Fig). The similarity between the genomes of strain zzj01 and other *E*. *faecalis* strains was higher than 98.73%. Strain zzj01 exhibited a close genetic relationship to *E*. *faecalis* strain SF28073 (GCF 014489455.1, 99.02% ANI), a clinical isolate from the urine sample of patients infected with vancomycin-susceptible *E*. *faecalis* [41]. Compared with the farthest and nearest strains, *E*. *faecalis* zzj01 shares over 2,000 genes with them but only possesses three unique genes (S1 Fig). This indicated a high degree of gene conservation between *E*. *faecalis* zzj01 and the other pathogenic strains.

**Fig 1:**
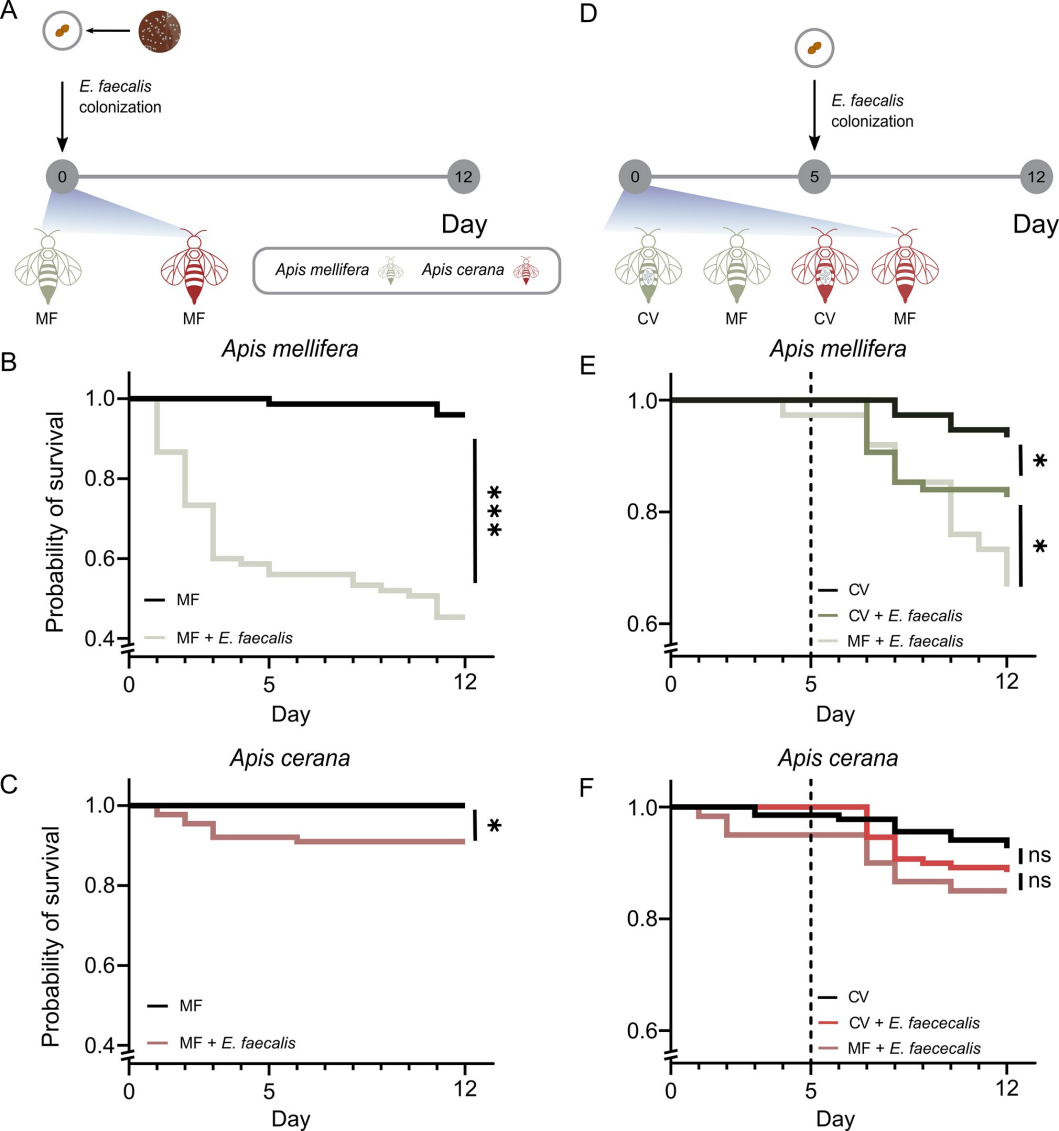
Survivorship of honeybees orally exposed to E. faecalis. (A) Schematic illustration of the experimental design for the treatment of MF honeybees. Survivorship of *A*. *mellifera* (B) and *A*. *cerana* (C) after oral exposure to *E*. *faecalis* was monitored and recorded each day for 12 days. (D) Schematic illustration of the experimental design for the treatment of CV honeybees. 5-day-old CV or MF bees were orally exposed to *E*. *faecalis*. Survivorship of *A*. *mellifera* (E) and *A*. *cerana* (F) were monitored and recorded each day for 7 days. n = 25 for each treatment group with three replicate experiments. *, P<0.05; **, P < 0.01; ***, P < 0.001 (Mantel-Cox test).

To determine the effect of *E*. *faecalis* zzj01 on MF bees, we conducted *in vivo* virulence assays and monitored the mortality rate of bees. We tested with both *A*. *cerana* and *A*. *mellifera* collected from mite-free brood frames. MF bees of *A*. *cerana* and *A*. *mellifera* were orally exposed to cell suspensions of *E*. *faecalis* or PBS (control group) in their food for 24 h and were fed sterilized sugar water and pollen for 12 days (Fig 1A). After 12 days of infection, *E*. *faecalis* only moderately altered the survivorship of *A*. *cerana* (Fig 1C), but the survival rate of *A*. *mellifera* decreased significantly (Fig 1B). These results indicate that *E*. *faecalis* zzj01 is virulent to *A*. *mellifera*, whereas *A*. *cerana* exhibits greater tolerance.

Since *E*. *faecalis* was identified during the generation of MF bees, and the hive bees seem unaffected, we wondered if the gut microbiota could protect the host against this pathogenic bacterium. We inoculated MF bees with conventional gut microbiota (CV) or PBS for 24 hours. After five days, bees were orally exposed to *E*. *faecalis* in their food for 24 hours (Fig 1D). In *A*. *mellifera*, CV bees infected with *E*. *faecalis* showed a significantly improved survival rate than MF bees (Fig 1E), suggesting that the gut microbiota protects the host against the deleterious effects of *E*. *faecalis*. However, *A*. *cerana* only showed a modest but non-significant improvement in survival (Fig 1F).

**Fig 2:**
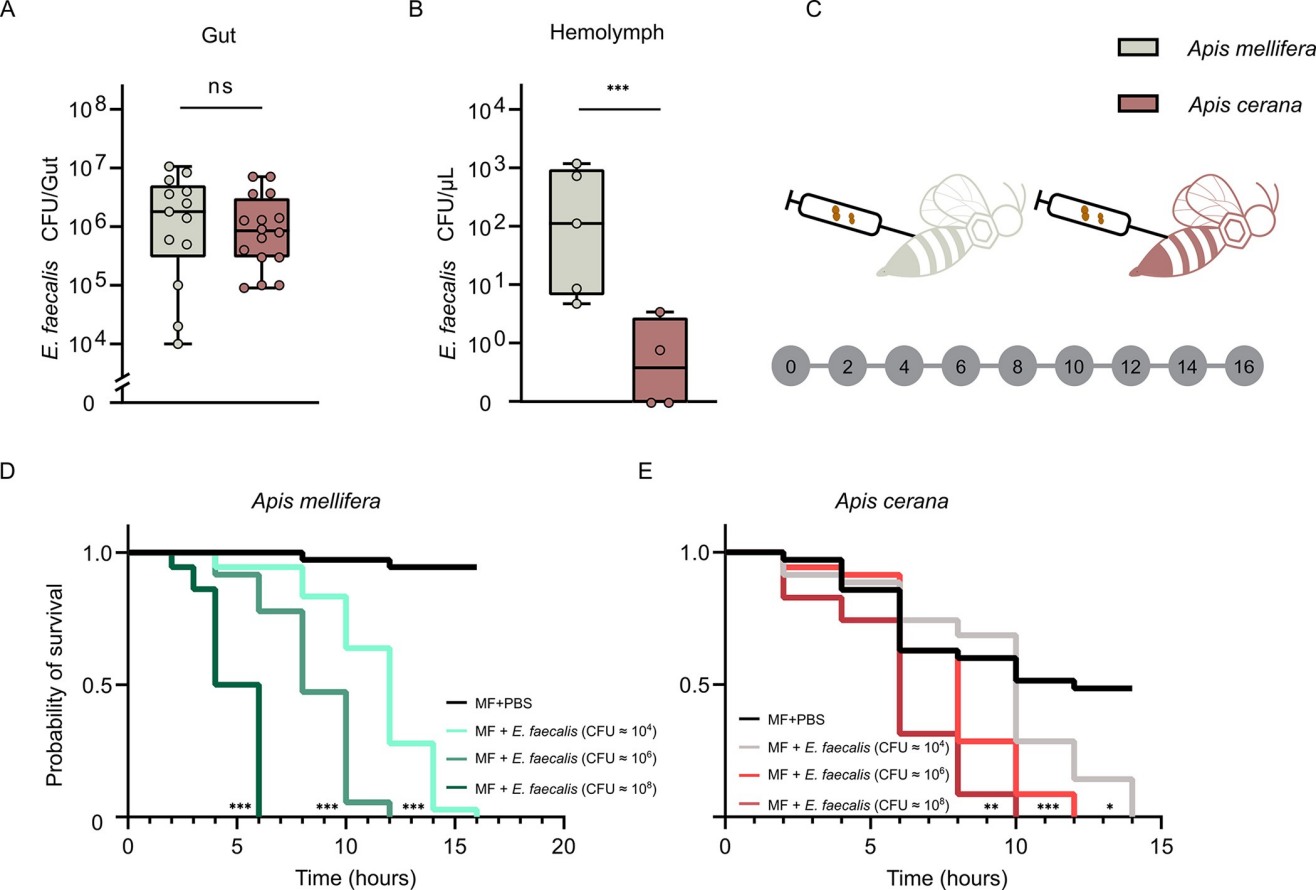
Hemolymph infection of E. faecalis leading to the death of honeybees. Absolute abundance of *E*. *faecalis* in the gut (A) and hemolymph (B) of *A*. *mellifera* and *A*. *cerana* in the MF group after 12 days post oral inoculation with *E*. *faecalis*. ***, P < 0.001; ns, not significance (Mann-Whitney *u* test). (C) Schematic illustration showing the experimental design for hemolymph injection of *E*. *faecalis*. Serial dilutions of *E*. *faecalis* in PBS were injected into the hemolymph of newly emerged MF honeybees. Survivorship of *A*. *mellifera* (D) and *A*. *cerana* (E) were monitored and recorded every 2 h for 16 h. n = 12 for each treatment group with three replicate experiments. *, *P*<0.05; **, *P* < 0.01; ***, *P* < 0.001 (Mantel-Cox test).

It has been reported that an opportunistic pathogen is lethal only after it enters the hemocoel of honeybees [23]. Since we did not observe high mortality in *A*. *cerana* after oral exposure, we collected gut and hemolymph samples of MF bees infected with *E*. *faecalis* (Day 12) to identify the number of infected bacteria. It shows that *A*. *cerana* and *A*. *mellifera* had similar *E*. *faecalis* colonization levels in the gut lumen (Fig 2A). However, the amount of *E*. *faecalis* in the hemolymph was considerably higher in *A*. *mellifera* than in *A*. *cerana* (Fig 2B). This indicates that the high mortality in *A*. *mellifera* may be due to the invasion of *E*. *faecalis* into the hemolymph via the gut. Next, we performed hemolymph injection experiments to further investigate the virulence of *E*. *faecalis* (Fig 2C). Notably, within 16 h, both *A*. *cerana* and *A*. *mellifera* perished after injection with *E*. *faecalis* suspensions at three different concentrations (OD_600nm_ = 1, 10^−2^, and 10^−4^) (Fig 2D and 2E). In addition, nearly half of *A*. *cerana* individuals that were injected with PBS died after 16 h, suggesting that puncture wounding may cause damage to *A*. *cerana*. These results indicate that infiltration of *E*. *faecalis* into the hemolymph is highly virulent to both *A*. *cerana* and *A*. *mellifera*, however, *A*. *cerana* may have developed specific mechanisms to impede the translocation of *E*. *faecalis* from the gut to the hemocoel.

### *E*. *faecalis* infection enhances the expression of AMP-encoding genes in *A*. *cerana* but not in *A*. *mellifera*

**Fig 3:**
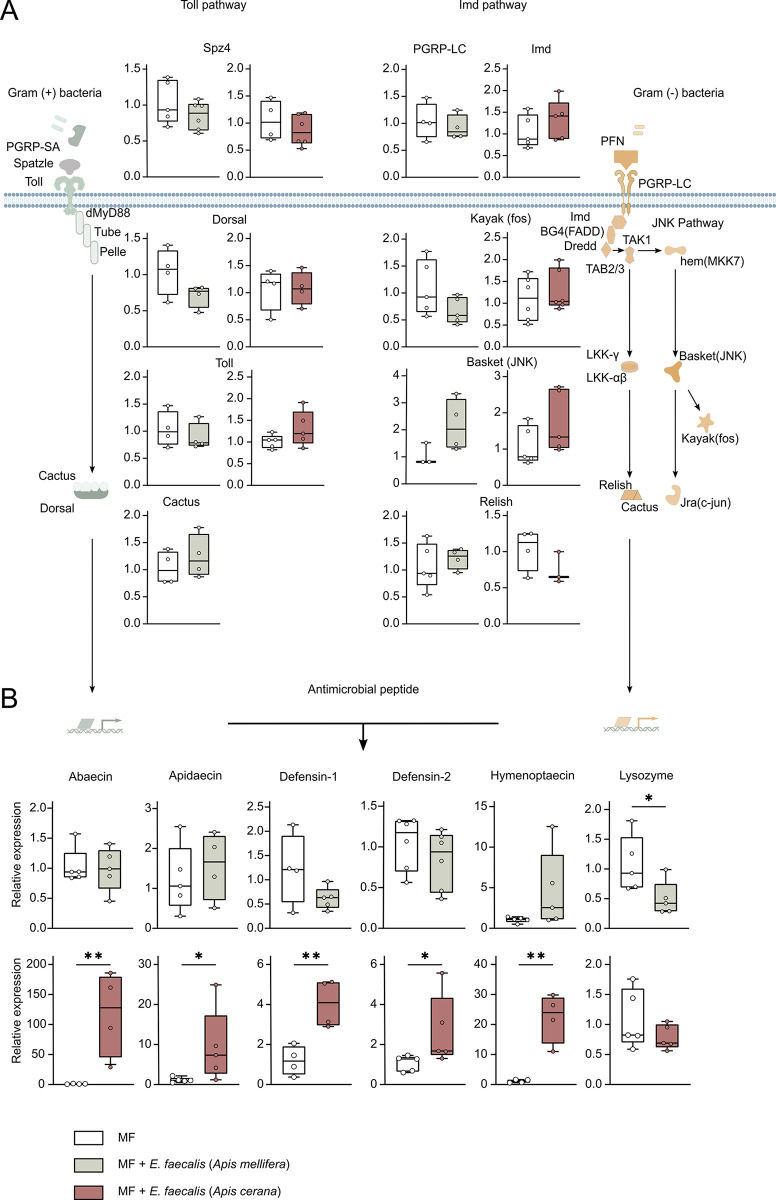
*A*. *mellifera and A*. *cerana* exhibit differential immune gene expression following *E*. *faecalis* inoculation. (A) The expression of immune regulatory genes in the Imd and Toll pathways did not show obvious differences between *A*. *mellifera* and *A*. *cerana*. (B) The expression of AMP genes was significantly increased in *A*. *cerana*. Gene expression was determined 24 h post infection. *, *P* < 0.05; **, *P* < 0.01 (Tukey honest method).

To explore the underlying mechanism of *A*. *cerana* against *E*. *faecalis*, we evaluated the relative expression of genes from the Toll and Imd pathways controlling the transcription of target genes encoding AMPs. We detected the receptors (*pgrp-lc*, *Spz4*, *toll*), the transcription factors (*relish*, *dorsal*), and their regulators (*kayak*, *basket*, *cactus*). No significant changes in the expression of these immune-related genes were observed between *E*. *faecalis* inoculated bees and the control groups in both *Apis* species (Fig 3A). Interestingly, all genes encoding AMPs except *lysozyme* showed significantly increased expression in *A*. *cerana* infected with *E*. *faecalis*. In particular, the expression of *hymenoptaecin* and *abaecin* increased up to 24-fold and 130-fold, respectively. However, in *E*. *faecalis*-infected *A*. *mellifera*, no AMP genes showed significantly increased expression.

### *Spz4* gene shows positive selection in *A*. *cerana*

**Fig 4:**
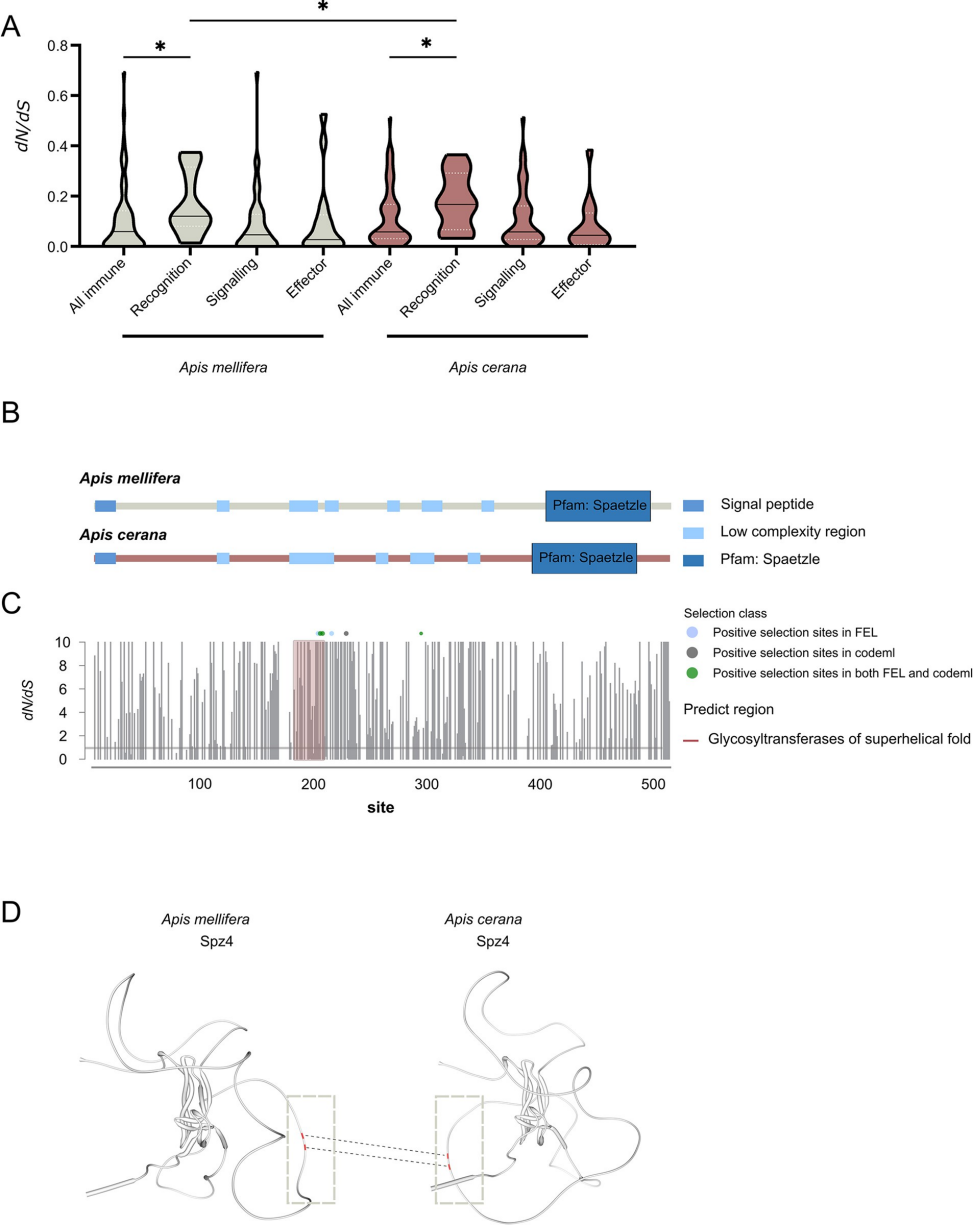
The positive selection of *Spz4* gene in *A*. *cerana*. (A) Violin plots showing *dN/dS* ratios for different categories of immune genes in *A*. *mellifera* and *A*. *cerana*. Black solid lines show medians of orthologus group values, and white dotted lines show the limits of the upper and lower quartiles. (B) The predicted structural domain of the Spz4 protein in *A*. *mellifera* and *A*. *cerana*. (C) Maximum likelihood estimations of *dN/dS* at each site of Spz4, together with estimated profile confidence intervals (if available). The *dN/dS* = 1 (neutrality) is depicted as a horizontal gray line. Boundaries between partitions (if present) are shown as vertical dashed lines. Predicted regions corresponding to glycosyltransferases are highlighted in red shadow (D). The predicted structure of the Spz4 protein in *A*. *mellifera* and *A*. *cerana* using the AlphaFold. The positive selection sites on the structure of Spz4 protein in *A*. *cerana* are represented in red, ranging from amino acids 188 to 206; the same sites are marked green in *A*. *mellifera*. *, *P* < 0.05 (Wilcoxon rank sum test).

Although the relative expression levels of AMP genes were significantly different between *A*. *mellifera* and *A*. *cerana*, no significant changes were observed in the gene expression of the receptors, the transcription factors, and their regulators of the Toll and Imd pathway (Fig 3). The coding sequence divergence of immune genes, especially for recognition receptors, is a major cause of differences in immune function between species [42]. Besides, selection on the amino acid composition of the immune-related genes has been an important part in the fight against pathogens by social insects [43]. Therefore, we tested for evidence of positive selection across immune-related genes in the genomes of different *Apis* species (*A*. *cerana*, *A*. *dorsata*, *A*. *florea*, *A*. *laboriosa*, *A*. *mellifera*) to determine which genes are subject to potential positive selection in *A*. *cerana*. First, the ratio of nonsynonymous (*dN*) to synonymous (*dS*) substitution rates (*dN/dS*) was calculated for the recognition, signaling, and effector genes in the immune pathways of *A*. *cerana*. It showed that the average *dN/dS* values of the recognition protein genes were higher than that of all immune genes in both the *A*. *cerana* and *A*. *mellifera* (Fig 4A and S2 Table). Interestingly, genes encoding recognition protein in *A*. *cerana* showed higher *dN/dS* ratios than those in *A*. *mellifera*, indicating that these genes have rapidly evolved in *A*. *cerana*.

To further investigate which genes have undergone adaptive evolution, we used the Codeml program of the *PAML* package to identify all immune-related genes showing signatures of positive selection in *A*. *cerana*. While many genes displayed elevated *dN/dS* values, only five immunological genes showed evidence of positive selection in *A*. *cerana*, including *Spz4*, *scavenger receptor class B member 1*, *TNF receptor-associated factor 4*, *agrin*, and *CTL7* (Table 1). Notably, *Spz4* has a significant role in the insect’s innate immune response [44], while the other four detected genes were not linked to the function of pathogen resistance.

**Table 1:** Genes under positive selection (using FDR < 0.05) on the branch to *A*. *cerana*.

Gene	Gene symbol	Classification	Total site	Positively selected sites
Spaetzle 4	LOC108002210	Toll pathway	514	204P, 206A, 231G, 295T
Scavenger receptor class B member 1	LOC108000323	scavenger receptors	407	2R, 3A, 4C
TNF receptor-associated factor 4	LOC107992533	TNF receptor-associated factor family	458	1M, 2D, 4A, 7I, 8T, 9D
Agrin	LOC108000879	serine protease inhibitors	506	3H, 5S, 7L, 11I
CTL7	LOC108000151	C-type lectin	206	2E, 4N, 5K, 11N, 12I, 14H, 17G

Next, we compared the protein sequence and predicted structural regions of Spz4 in *A*. *cerana* and *A*. *mellifera*. It showed that the main sequence differences were located between amino acids 190 and 220 (Fig 4B). In *A*. *cerana*, amino acid 188 to 206 of Spz4 corresponded to the glycosyltransferases with an α–α superhelical fold. Glycosyltransferases are involved in forming mucus proteins, which are important to the insect intestinal physical defense systems against pathogen invasion [45]. In contrast, the corresponding region in *A*. *mellifera* was predicted to be disordered without a stable three-dimensional structure in the Spz4 protein.

To verify the positively selected sites within the *Spz4* gene, we used the Datamonkey web server and identified five distinct sites (amino acid positions: 202Q, 204P, 206A, 216S, 295T) exhibiting positive selection. Interestingly, three of these sites (amino acid positions 202Q, 204P, 206A) were clustered within the region corresponding to glycosyltransferases (amino acids 188 to 206) (Fig 4C). Notably, the positions at 204P and 206A were consistent with the positively selected sites identified in *Spz4* using Codeml (Table 1). In addition, the predicted protein structure of Spz4 showed difference between *A*. *cerana* and *A*. *mellifera* (Fig 4D). Our findings suggested that the changes in amino acids will likely lead to structural changes in Spz4, induce the synthesis of glycosyltransferases, and finally result in improved immune responses in *A*. *cerana*.

### *Spz4* plays a significant role in *A*. *cerana’s* developing immunity

**Fig 5:**
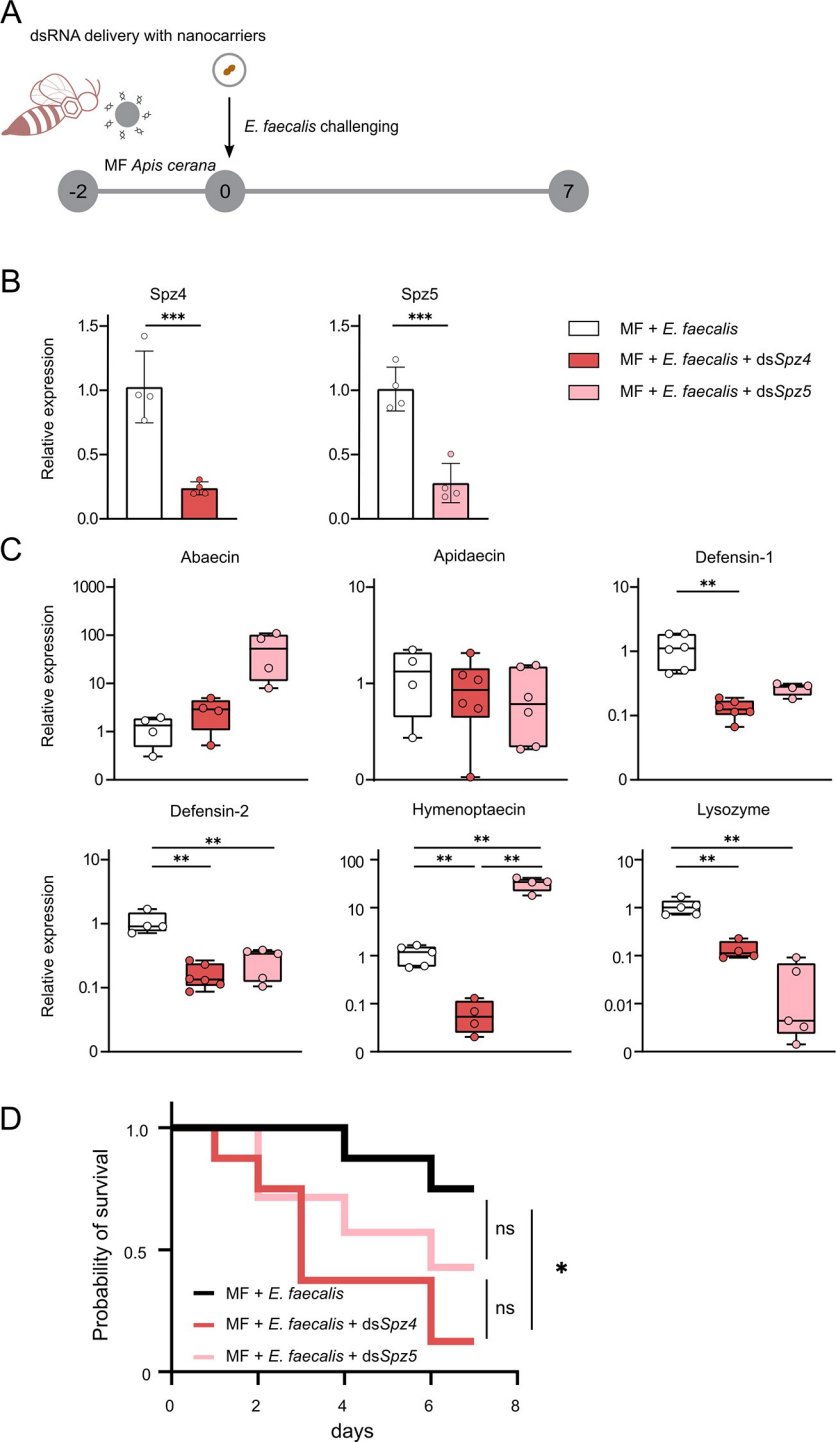
The immune response against *E*. *faecalis* in RNAi-treated *A*. *cerana*. (A) Schematic illustration of the experimental design. Knockdown of *Spz4* or *Spz5* gene expression in *A*. *cerana* was achieved by feeding nanoparticle-mediated dsRNA. Bees were then inoculated with *E*. *faecalis*. (B)The expression of *Spz4* and *Spz5* in *A*. *cerana* after oral treatment with ds*Spz4* or ds*Spz5* for two days. The expression level of AMP genes in the gut (C) and the survival rate (D) of *A*. *cerana* in the control (MF) and RNAi (MF + ds*Spz4* or MF + ds*Spz5*) groups infected with *E*. *faecalis*. **, *P* < 0.01; ***, *P* < 0.001 (Tukey honest method). *, *P*<0.05; ns, not significance (Mantel-Cox test).

Our results showed that the *Spz4* gene was undergoing positive selection in *A*. *cerana*. In honeybees, six spätzle homologues (*Spz1–6*) have been identified (*Spz1–6* in *A*. *mellifera*; *Spz1* and *Spz3-5* in *A*. *cerana*) [46]. Among them, *Spz4* and *Spz5* have been shown to regulate the production of AMPs against microbial infections [47,48]. However, no evidence of positive selection was observed in the *Spz5* gene. Therefore, we chose *Spz5* as a negative control to verify the functions of *Spz4* in *A*. *cerana*. Here, we used the nanoparticle-mediated dsRNA delivery system to facilitate the RNAi silencing efficiency of *Spz4* and *Spz5* in *A*. *cerana* (Fig 5A) [49]. By feeding ds*Spz4* or ds*Spz5* to MF bees, the mRNA transcript levels of *Spz4* and *Spz5* decreased by about 70% after three days (Fig 5B). Then, we challenged the silenced and the control groups of bees with *E*. *faecalis*. In *Spz4*-silenced bees, the expressions of *defensin-1*, *defensin-2*, *hymenoptaecin*, and *lysozyme* were significantly decreased after *E*. *faecalis* infection (Fig 5C), and *hymenoptaecin* was the most significantly down-regulated AMP. In *Spz5*-silenced bees, *defensin-2* and *lysozyme* were also considerably reduced, while *hymenoptaecin* was significantly increased. Notably, the expression of *lysozyme* was not induced in *E*. *faecalis*-infected *A*. *cerana* without RNAi (Fig 3B). These results indicated that *hymenoptaecin* regulated by *Spz4* might be the key AMP in *A*. *cerana* to defend against *E*. *faecalis*. Finally, we evaluated the survival of *Spz4-* or *Spz5*-silenced *A*. *cerana* infected with *E*. *faecalis* for seven days (Fig 5A). As expected, ds*Spz4*-fed bees were significantly more susceptible to *E*. *faecalis* and had a higher lethality than ds*Spz5*-fed bees (Fig 5D). Altogether, these data confirm the significance of *Spz4* in the innate immune defense against *E*. *faecalis*.

## Discussion

In this study, we isolated *E*. *faecalis* strains during the generation of MF bees obtained from mite-infected colonies. These *E*. *faecalis* strains cause a lethal infection in *A*. *mellifera* but are not virulent to *A*. *cerana*. Compared with *A*. *mellifera*, several immune-related genes have undergone positive selection in *A*. *cerana*. Specifically, *Spz4* in *A*. *cerana* regulated the production of AMPs and was essential to protect *A*. *cerana* against *E*. *faecalis*. Our findings extend previous knowledge of *V*. *destructor*-associated bacterial pathogens and highlight the variation in the immune system between different honeybee species.

*V*. *destructor* has attracted long-term attention from researchers, since it can parasitize honeybees, feed on their hemolymph [50], and spread various viruses [8] that can be detrimental to honeybee colonies. Except for viruses, bacteria have been found in the open wounds of honeybee pupae and adults, where mouthparts of the mite have penetrated the membrane [22,51]. However, little is known about the species of these bacteria and their role in *V*. *destructor*-induced damage to bees. Based on 16S rRNA sequencing analysis, a wide variety of bacterial taxa has been detected in *V*. *destructor*, including species of *Morganella*, *Enterococcus*, and *Arsenophonus* [16,52]. *E*. *faecalis* is one of the most abundant OTUs associated with *V*. *destructor* [15–18]. We found that worker bees from mite-infested colonies were infected with *E*. *faecalis*. The isolated *E*. *faecalis* strain was strongly pathogenic to *A*. *mellifera*. These findings suggest that *V*. *destructor* can also transmit pathogenic bacteria and lead to the death of the host. Moreover, we found that oral treatment of *A*. *mellifera* with *E*. *faecalis* resulted in substantial proliferation of this bacterium in the hemolymph. In most animals, opportunistic pathogens are virulent only when present in the hemocoel, such as *S*. *marcescens* in *A*. *mellifera* [23,24] and *E*. *faecalis* in Tobacco hornworm (*M*. *sexta*) [53]. Thus, the pathogenicity of *E*. *faecalis* in honeybees may be correlated with the ability of the strain to penetrate the gut wall and enter the hemolymph, causing death. Alternatively, wounds from mite bites could enable mite-carried bacteria, especially *E*. *faecalis*, to access the hemolymph directly, which is mimiced in our hemolymph injection experiments.

Although oral treatment with *E*. *faecalis* was deadly to *A*. *mellifera*, *A*. *cerana* could resist and survive the infection. As the original host of *V*. *destructor*, *A*. *cerana* has developed many defenses against mites, including social immunity and hygienic behavior [54]. In addition, genomic and transcriptomic analyses reveal that *A*. *cerana* has favored the generation of more variable AMPs as protection against pathogens, suggesting the evolution of specific innate immunity in long-term symbiotic associations with parasitic mites [13,14]. Our results show that the gene expression of most AMPs was significantly elevated in *A*. *cerana* than in *A*. *mellifera*, indicating that *A*. *cerana* exhibits a more robust immune response to mite-associated *E*. *faecalis*. Consistently injecting a toxic *Varroa* protein from the saliva of *Varroa* mites induces differential expression patterns of AMPs in the larvae of *A*. *mellifera* and *A*. *cerana* [55].

Honeybee antibacterial immunity relies on the Toll and Imd pathways to regulate the production of AMPs during pathogen infection in honeybees to defend against pathogenic bacteria [56,57]. However, we did not observe significantly different expression levels of genes in the Toll and Imd signaling pathway between *A*. *cerana* and *A*. *mellifera*. In social insects, such as ants, termites, and social bees, immune genes exhibit high evolutionary rates under the high risk of pathogen transmission due to close social contact of many individuals in large colonies [43]. Genes in the Toll pathway had a higher rate of nonsynonymous mutations between *A*. *cerana* and *A*. *mellifera* [58], which favored the generation of more variable AMPs in *A*. *cerana* as protection against pathogens [14]. We found that *Spz4*, a Toll receptor ligand, showed positive selection in *A*. *cerana* (Fig 4), which may lead to changes in the corresponding immune proteins, such as AMPs [8,59]. Our results suggest that the innate immunity of *A*. *cerana* has undergone adaptive evolution during the long-term coevolutionary interaction with *E*. *faecalis*-carrying *V*. *destructor*.

The Spz is a gene family encoding regulatory proteins in the insect immune system, which is critically involved in embryonic development and the innate immune response of insects [20,44,48,60]. In mosquitoes and mealworms, *Spz4* is required for Toll signaling activation and AMP production to defend against bacteria and fungi [47,61]. Fungi infection can suppress host immunity by silencing the expression of *Spz4* in the fat body of mosquitoes [61]. *Spz4* knock-down can downregulate the expression level of AMPs and significantly reduce mealworm larval survival against bacterial pathogens [47]. Although we did not observe significant changes in *Spz4* gene expression after *E*. *faecalis* infection, the protein sequence and predicted structure of Spz4 are different between *A*. *cerana* and *A*. *mellifera*. Spz paralogues may have various signaling activities. *Drosophila* Spz1 can stimulate gambicin moderately in Aag2 cells, whereas *Aedes aegypti* Spz1C cannot due to structural changes upon diversification [62]. We found that the positively selected sites of *Spz4* in *A*. *cerana* correspond to the glycosyltransferases, which are involved in mucus layer formation and the physical defense system in insects [45,63]. Therefore, we speculate that the *Spz4* gene in *A*. *cerana* has acquired new functions under positive selection, and affects honeybee immune response to *E*. *faecalis*.

In *Drosophila*, Spz5 has also been reported to function as a ligand for Toll receptor and regulate the production of AMPs [48,64]. However, the predicted cystine knot domains indicate that Spz4 and Spz5 are not closely related [48]. RNAi-mediated knockdown of Spz4 in adult flies can disrupt Toll pathway activation, whereas Spz5 is not required to induce AMPs [64,65]. Our RNAi experiments indicate that the knockdown of Spz4 or Spz5 can modulate the expression of certain AMPs in *A*. *cerana* following *E*. *faecalis* infections. Hymenoptaecin may be the critical AMP against *E*. *faecalis*, as it is oppositely regulated in Spz4-silenced and Spz5-silenced bees. In addition, Spz4-silenced bees showed a significantly lower survival rate than Spz5-silenced bees after challenging with *E*. *faecalis*. Collectively, our findings support that Spz4 plays a crucial role in protecting *A*. *cerana* from *E*. *faecalis* infection, probably by regulating the expression of hymenoptaecin.

In conclusion, our research indicates the high virulence of bacteria associated with *Varroa* mites to *A*. *mellifera*, highlighting the crucial role of the *Spz4* gene in *A*. *cerana* immunity. Considering the long-term coexistence between *A*. *cerana* and *V*. *destructor*, it is implied that *A*. *cerana* has developed specific immunity mechanisms to defend against pathogens associated with *V*. *destructor*. Future work should be conducted to verify the transmission of *E*. *faecalis* from *V*. *destructor* to honeybees. Furthermore, investigations of the impact of honeybee gut microbiota on *E*. *faecalis* may provide new insights into the mechanisms underlying *V*. *destructor*-induced honeybee colony mortality and potential strategies for improving honeybee colony health.

## Supporting information

S1 Fig Phylogenetic analysis of *E*. *faecalis*. (A) Phylogenetic tree of *E*. *faecalis* strains. The tree was constructed using the genome sequences of all available *E*. *faecalis* strains and a genome of *Melissococcus plutonius* (GCF_003966875.1) collected from the Refseq database (June 2021). Bootstrap values are represented by circles at each node. (B) The Venn diagram shows the number of genes unique or shared among *E*. *faecalis* zzj01 and the nearest and farthest strains from *E*. *faecalis* zzj01.
(TIF)

S1 Table qPCR primer sequences for gene expression analysis. (PDF)

S2 Table The immune gene of *dN/dS* value was estimated by Codeml (Model 1). Positively selected genes are highlighted in the items.
(PDF)
