# Supplementary figures and images for: Toll receptor ligand Spätzle 4 responses to the highly pathogenic Enterococcus faecalis from Varroa mites in honeybees
Source: PLoS Pathog. 2023 Dec 27;19(12):e1011897. doi: 10.1371/journal.ppat.1011897 (PMC10775982; doi:10.1371/journal.ppat.1011897)

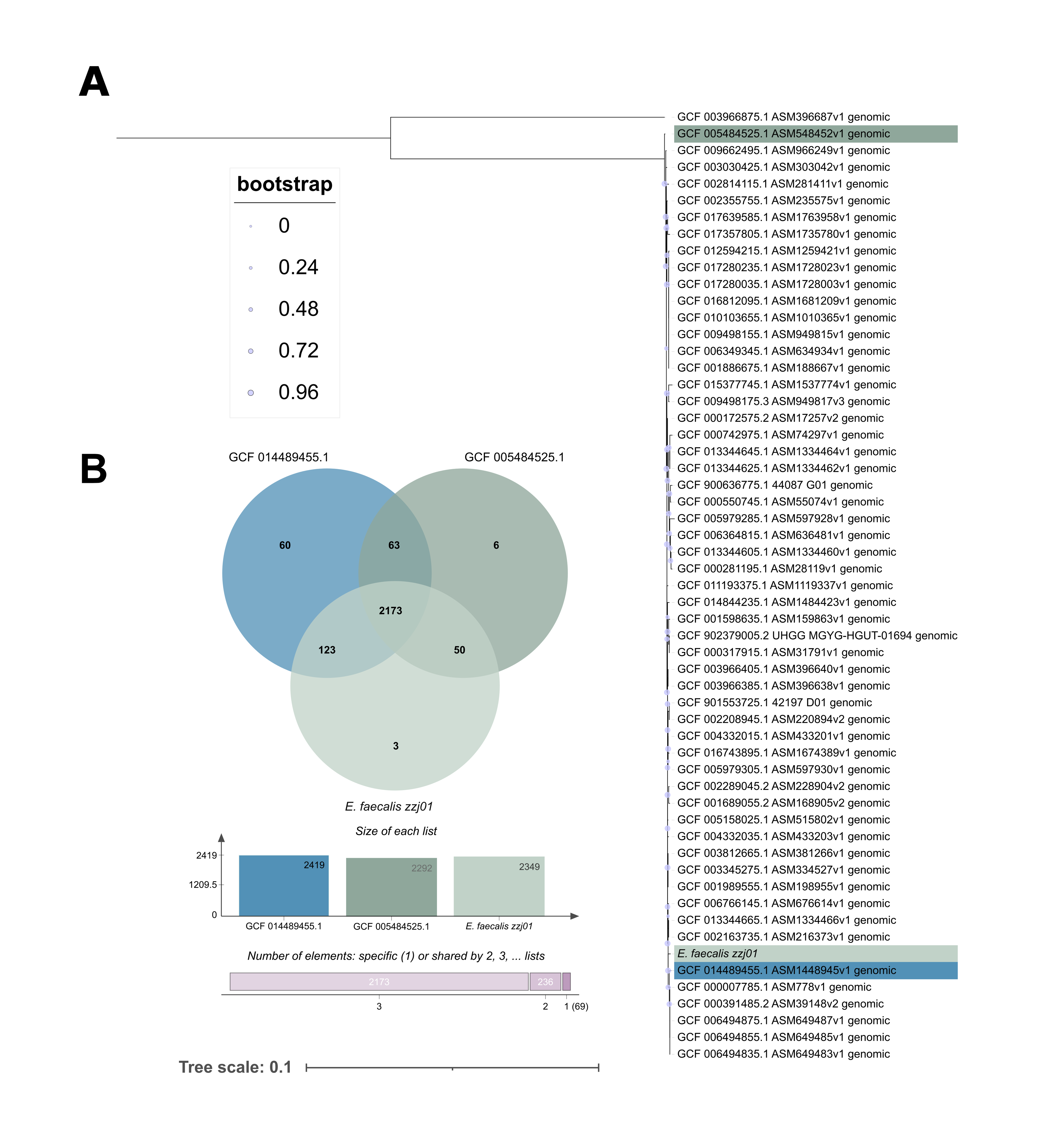

Supplement: S1 Fig — (A) Phylogenetic tree of E. faecalis strains. The tree was constructed using the genome sequences of all available E. faecalis strains and a genome of Melissococcus plutonius (GCF_003966875.1) collected from the Refseq database (June 2021). Bootstrap values are represented by circles at each node. (B) The Venn diagram shows the number of genes unique or shared among E. faecalis zzj01 and the nearest and farthest strains from E. faecalis zzj01. (TIF) [file ppat.1011897.s001.tif]
