# Supplementary material for: Toll receptor ligand Spätzle 4 responses to the highly pathogenic Enterococcus faecalis from Varroa mites in honeybees
Source: PLoS Pathog. 2023 Dec 27;19(12):e1011897. doi: 10.1371/journal.ppat.1011897 (PMC10775982; doi:10.1371/journal.ppat.1011897)
Supplement: S1 Table — (PDF) [file ppat.1011897.s002.pdf]

Supplemental Tables

S1 Table. qPCR primer sequences for gene expression analysis

Species	Gene	Reference	Forward	Reverse
A. mellifera	*Abaecin*	[1]	TCGGATTGAATGGTCCCTGAC	ATCTTCGCACTACTCGCCAC
A. mellifera	*Apodaca*	[1]	GTAGGTCGAGTAGGCGGATCT	TTTTGCCTTAGCAATTCTTGTTG
A. mellifera	*Cactus-1*	[1]	CTATCGTGGAGAAACTGCGTAT	TCAGGAAGTGGTTCTGGTATTG
A. mellifera	*Cactus-2*	[1]	ATCAGACGGCTCTGCTCTAT	TCGTCTTCGTCAGTGGTATCT
A. mellifera	*Dorsal*	[1]	AGAGATGGAACGCAGGAAAC	TGACAGGATATAGGACGAGGTAA
A. mellifera	*Dredd*	[1]	GCGTCATAAAGAAAAAGGATCA	TTTCGGGTAATTGAGCAACG
A. mellifera	*Hymenoptaecin*	[1]	GTCGTCCATCCTTGGACATT	TTTCCCAAACTCGAATCCTG
A. mellifera	*PGRP-LC*	[1]	TCCGTCAGCCGTAGTTTTTC	CGTTTGTGCAAATCGAACAT
A. mellifera	*Relish*	[1]	GGAGCTGATCCAAATCGAAC	AGTGGCATCCATCCATCATT
A. mellifera	*RPS18*	[1]	AGGTGTTGGTCGTCGTTAT	CATTCTCCAGCACGCTTAT
A. mellifera	*Toll*	[1]	TAGAGTGGCGCATTGTCAAG	ATCGCAATTTGTCCCAAAAC
A. mellifera	*Defensin-1*	[2]	TGCGCTGCTAACTGTCTCAG	AATGGCACTTAACCGAAACG
A. mellifera	*Defensin-2*	[3]	GCAACTACCGCCTTTACGTC	GGGTAACGTGCGACGTTTTA
A. mellifera	*Lysozyme*	[3]	ACACGGTTGGTCACTGGTCC	GTCCCACGCTTTGAATCCCT
A. mellifera	*Kayak*	[4]	CGACAGATCCGCAGAGAAAG	CCTGTTGCAGCTGTTGTATC
A. mellifera	*Basket*	[4]	AGGAGAACGTGGACATTTGG	AGGAGAACGTGGACATTTGG
A. cerana	*actin*	[5]	CTCACAGTGTTCGCAACTCG	CGAAACCGGCTTTGCACATA
A. cerana	*abaecin*	[5]	AGAGTTTGATCCTGGCTCAG	CTGCTGCCTCCCGTAGGAGT
A. cerana	*apidaecin*	[5]	CCAGATCCGCCTACTCAACC	GGTTTAGCTTCACGGCGTAG
A. cerana	*defensin1*	[5]	AGCCACTTGAGCATCCTGAG	CCGTTCTTGCAATGACCTCC
A. cerana	*defensin2*	[5]	TTTCGCGATTCTCGTCGCTA	TGTCGTAGCAGTAGCGGTTC
A. cerana	*hymenoptaecin*	[5]	CGTGTTGGTTGTCTTCTGCG	CACCATAGGCATCTCCCGTC

A. cerana	*relish*	[5]	TGAAGCTGGTGCATGTGTTG	CCTGCTTTTGCTGCAAGATGT
A. cerana	*dorsal*	[5]	TTTATCACGATTGTAGATGCTGC	GGAGAAGTTGTTGCCATCGG
A. cerana	*basket*	[5]	AGGAGAACGTGGACATTTGG	AATCCGATGGAAACAGAACG
A. cerana	*Imd*	[5]	TGTTAACGACCGATGCAAAA	CATCGCTCTTTTCGGATGTT
A. cerana	*toll*	[5]	TCGATGTCCAACGGAGCAAA	ACTTTCACAACGAAGGCCGA
A. cerana	*domeless*	[5]	TTGTGCTCCTGAAAATGCTG	AACCTCCAAATCGCTCTGTG
A. cerana	*kayak*	[5]	CGACAGATCCGCAGAGAAAG	CCTGTTGCAGCTGTTGTATC
A. cerana	*catalase*	[6]	GTCTTGGCCCGAACAATTTG	CATTCTCTAGGCCCACCAAA

References

1. Horak RD, Leonard SP, Moran NA. Symbionts shape host innate immunity in honeybees. Proc R Soc B Biol Sci. 2020;287: 20201184. doi:10.1098/rspb.2020.1184

2. Daisley BA, Pitek AP, Chmiel JA, Al KF, Chernyshova AM, Faragalla KM, et al. Novel probiotic approach to counter *Paenibacillus larvae* infection in honey bees. ISME J. 2020;14: 476–491. doi:10.1038/s41396-019-0541-6

3. Daisley BA, Pitek AP, Chmiel JA, Gibbons S, Chernyshova AM, Al KF, et al. *Lactobacillus* spp. attenuate antibiotic-induced immune and microbiota dysregulation in honey bees. Commun Biol. 2020;3: 1–13. doi:10.1038/s42003-020-01259-8

4. Tesovnik T, Zorc M, Gregorc A, Rinehart T, Adamczyk J, Narat M. Immune gene expression in developing honey bees (*Apis mellifera* L.) simultaneously exposed to imidacloprid and *Varroa destructor* in laboratory conditions. J Apic Res. 2019;58: 730–739. doi:10.1080/00218839.2019.1634463

5. Wu Y, Zheng Y, Chen Y, Chen G, Zheng H, Hu F. *Apis cerana* gut microbiota contribute to host health though stimulating host immune system and strengthening host resistance to *Nosema ceranae*. R Soc Open Sci. 7: 192100. doi:10.1098/rsos.192100

6. Chao Y, Wang C, Jia H, Zhai N, Wang H, Xu B, et al. Identification of an *Apis cerana cerana* MAP kinase phosphatase 3 gene (*AccMKP3*) in response to environmental stress. Cell Stress Chaperones. 2019;24: 1137–1149. doi:10.1007/s12192-019-01036-5
