# Supplementary material for: Toll receptor ligand Spätzle 4 responses to the highly pathogenic Enterococcus faecalis from Varroa mites in honeybees
Source: PLoS Pathog. 2023 Dec 27;19(12):e1011897. doi: 10.1371/journal.ppat.1011897 (PMC10775982; doi:10.1371/journal.ppat.1011897)
Supplement: S2 Table — Positively selected genes are highlighted in the items. (PDF) [file ppat.1011897.s003.pdf]

Supplemental Tables

S2 Table. The immune gene of *dN/dS* value was estimated by Codeml (Model 1).

Positively selected genes are highlighted in the items.

Gene family	AC gene id	AM (*dN/dS*)	AC (*dN/dS*)
**Recognition**			
PGRP-SA	108004045	0.373988	0.364077
peptidoglycan-recognition protein SC2	108004046	0.106223	0.168038
peptidoglycan-recognition protein LC-like	107999442	0.335848	0.303464
beta-1,3-glucan-binding protein	107999037	0.360785	0.15665
beta-1,3-glucan-binding protein 1	107993481	0.144236	0.255875
galectin-8	107994825	0.153047	1.20366
Fibrinogen-domain	107999732	0.0147401	0.0321197
scavenger receptor class B member 1	108000324	0.255866	0.323534
scavenger receptor class B member 1	108000323	0.0135219	0.209226
scavenger receptor class B member 1-like	107998966	0.114999	0.165698
scavenger receptor class B member 1-like	107998401	0.091877	0.103851
scavenger receptor class B member 1	107999764	0.0760734	0.0540138
protein bark beetle （SRCR domain）	108003374	0.125382	0.044167
**Signaling**			
CLIP-associating protein 1-A	108001004	0.00651003	0.0188924
proclotting enzyme	108002100	0.107926	0.137475
serine protease snake	108002098	0.183534	0.287815
serine protease inhibitor 88Ea	107993022	0.0621342	0.0819824
toll-like receptor 6	108000709	0.14802	0.0578013
toll-like receptor 6	108001858	0.230469	0.230259
toll-like receptor Tollo	108001172	0.0308253	0.0001
toll-interacting protein	107997044	0.0322739	0.0278991
toll-like receptor 4	107996866	0.0001	0.160302
artichoke	107998162	0.025478	0.0767973
sterile alpha and TIR motif-containing protein 1	107997290	0.0521897	0.375635
mini-chromosome	107997479	0.13901	0.162598
dorsal root ganglia homeobox protein	108003366	0.0001	0.153712
nuclear factor NF-kappa-B p100 subunit	107995490	0.222279	0.221524
phenoloxidase 1	108004136	0.0887741	0.0575564
membrane-associated protein Hem	108001136	0.0001	0.0001
JNK-interacting protein 3	107993771	0.0454203	0.0500499
JNK-interacting protein 1	107999316	0.0160945	0.0462117
dual specificity protein phosphatase 15	108002085	0.0001	0.0364175
transcription factor kayak	107997622	0.0001	0.0001
PRKC apoptosis WT1 regulator protein	107994477	0.0001	0.28234
apoptosis-inducing factor 3	107998476	0.111058	0.111019
apoptosis-inducing factor 1, mitochondria	107994937	0.0586742	0.0547875
apoptosis inhibitor 5	108000380	0.0001	0.0001
apoptosis-resistant E3 ubiquitin protein ligase 1	107996079	0.0001	0.0001
cell division cycle and apoptosis regulator protein 1	108000402	0.0457711	0.0413396

apoptosis regulatory protein Siva-like	108000282	0.113261	1.08855
anamorsin homolog	108003921	0.147575	0.268725
programmed cell death protein 6	108002730	0.0001	0.0955091
calpain-C	107999437	0.0001	0.0169598
caspase-1	107998622	0.0907679	0.0393119
protein lifeguard 1	107998291	0.0001	0.0268008
tyrosine-protein kinase hopscotch	107997913	0.0468087	0.0216028
imd	107998367	0.335679	0.271041
lap2	107995066	0.123578	0.0533615
protein spaetzle 3	107995066	0.123578	0.0533615
protein spaetzle 4	108002210	0.0548925	0.13974
protein spaetzle 5-like	107995531	0.24596	0.148678
uncharacterized LOC108001130（SPZ6）	108001130	0.00800723	0.0294888
MyD88-A	107997261	0.145689	0.120779
protein Tube	107996597	0.336584	0.122878
serine/threonine-protein kinase pelle	108001969	0.277012	0.0458983
protein pellino	107995854	0.0001	0.0001
dorsal root ganglia homeobox protein	108003366	0.0001	0.153712
TNF receptor-associated factor 4	107992533	0.0001	0.349451
TNF receptor-associated factor 6-like	107996279	0.0609658	0.511632
agrin	108000879	0.0001	0.191588
follistatin-related protein 5	107995521	0.0461954	0.0152436
uncharacterized LOC108000744	108000744	0.691627	0.330273
caspase-1	107998622	0.0907679	0.0393119
baculoviral IAP repeat-containing protein 6	107997826	0.0160085	0.0217962
**Effector**			
superoxide dismutase [Cu-Zn]	108000927	0.0001	0.0001
copper transport protein ATOX1	108002878	999	0.0001
phenoloxidase 1	108004136	0.0887741	0.0575564
phenoloxidase-activating factor 2	108000782	0.415262	0.133173
dual oxidase	107993848	0.0271269	0.00734858
probable phospholipid hydroperoxide glutathione peroxidase	107994450	0.02669	0.027481
probable phospholipid hydroperoxide glutathione peroxidase	108003552	0.0001	0.179853
MD-2-related lipid-recognition protein	108004050	0.166838	0.0507847
LOC107999732	107999732	0.0147401	0.0321197
CTL7	108000151	0.0001	0.382913
collectin-11	107998589	0.0001	0.0001
L-selectin	108003381	0.0001	0.0001
protein bark beetle	108003374	0.125382	0.044167
pentraxin domain-containing protein 1	107997239	0.0842397	0.0397371
oxidized low-density lipoprotein receptor 1	108000538	0.0600304	0.0719715
Defensin-1	107993803	0.525262	0.142264
